# Editorial: Phytohormones and the Regulation of Stress Tolerance in Plants: Current Status and Future Directions

**DOI:** 10.3389/fpls.2017.01871

**Published:** 2017-10-31

**Authors:** Vijay P. Singh, Sheo M. Prasad, Sergi Munné-Bosch, Maren Müller

**Affiliations:** ^1^Department of Botany, Govt. Ramanuj Pratap Singhdev Post Graduate College, Baikunthpur, India; ^2^Ranjan Plant Physiology and Biochemistry Laboratory, Department of Botany, University of Allahabad, Allahabad, India; ^3^Departament de Biologia Vegetal, Facultat de Biologia, Universitat de Barcelona, Barcelona, Spain

**Keywords:** phytohormones, abiotic stress tolerance, heavy metal, salinity, drought stress, temperature

Stresses, either abiotic or biotic, severely affect agricultural productivity and world agriculture is facing the challenge of increasing the productivity in order to pace up with the increasing food demand of the growing population. These stresses have been reported to negatively affect the biomass and yield of crops up to 70% (Parihar et al., 2015). The tremendous problem of matching food demands with increasing human population has drawn attention of scientists worldwide, and in order to solve the problem of a decline in productivity due to biotic and abiotic stresses, several experiments are being carried out all over the world. Several approaches, such as developing hybrid varieties, development of chemical priming and use of genetically modified crops are being followed. This research topic, which compiled 7 reviews and 20 research articles aimed at collecting our knowledge about the effect of abiotic stresses on transcriptomics, plant response to combined stress factors, as well as the regulatory role of several compounds in stress conditions.

Review articles covered our current knowledge about the involvement of different hormones in growth and development processes and their role under stress conditions, including more novel aspects, such as hormone cross-talk. Vishwakarma et al. reviewed details about the abscisic acid signaling and its involvement under abiotic stresses. Skubacz et al. reviewed the role of ABI5 in plant development and response to abiotic stress including its cross-talk with other phytohormones. The role of brassinosteroids, strigolactones, jasmonates, and other phytohormones was also compiled in reviews by Ahmad et al., Rajewska et al., Oracz and Karpinski, Phukan et al., and Mishra et al.

Research articles included a number of aspects of plant responses to abiotic stresses, from molecular mechanisms of plant stress tolerance to other approaches used to mitigate these stresses in crop plants. Wang et al. studied the involvement of several transporters e.g., *cadmium-transporting ATPase, plant cadmium resistance 4, ABC transporters*, and *metal chelators*, among these, that were either up- or down-regulated under Cd and Zn treatments. Some other genes involved in oxidation-reduction (redox) metabolism of metabolites also tend to play important roles in detoxification processes. Similar to this, Zhu et al. showed that *MEB2*, a vacuolar gene, was involved in storing and detoxifying excess iron in *Brassica napus*. Another work performed by Yang et al. showed that high temperature stress leads to an accumulation of *NtMYC2a* which activates the synthesis of nicotine and jasmonic acid. Similarly, Huang and Wang, Ksouri et al., Cao et al., and Wu et al. also showed that transcriptional factors influence plants resistance under stress conditions.

Hormones are not only involved in regulating growth and developmental processes, but their exogenous applications may also help to improve the plant yield. Li et al. observed improved photosynthetic activity, nitrogen metabolism and amino acid production by applying brassinosteroids to *Camellia sinensis*. Similarly, Gondor et al. showed an improvement in flavonoid biosynthesis after exogenous application of salicylic acid. Li et al. showed that synergistic priming of maize seeds with salicylic acid and H_2_O_2_ improves hormone metabolism and their transduction, stimulating energy supply and antioxidant systems, which in turn help maize seedlings to tolerate chilling stress. Studies by Agarwal et al., Sharma et al., Per et al., Cheng et al., Gruszka et al., and Patanun et al. also proved the involvement of brassinosteroids, salicylates, and jasmonates under a number of abiotic stresses. In general, the common mechanism of action suggested in these studies was up-regulation of genes associated with antioxidant protection, expression of specific transcription factors, and activation of specific responsive pathways, including hormone homeostasis.

Among different exogenous mitigants, not only the role of hormones was explored, but some research articles also studied the impact of other mitigants like H_2_S and glycerol. H_2_S was found to regulate changes in polyamine and sugar metabolism and thus providing drought tolerance (Chen et al.), while glycerol application provided tolerance against powdery mildew disease by inducing expression of pathogenesis-related genes (Li et al.). Other than these, research articles also explored the possible involvement of E-subgroup pentatricopeptide repeat protein family under abiotic stress (Liu et al.). Furthermore, He et al. showed that phytochrome B regulates expression of *DREB1* gene to enhance cold tolerance.

Overall, these articles give a clear picture of ongoing dynamic research on phytohormones and other compounds in preventing negative effects of abiotic stresses. The impact of several stresses on the transcriptome, phytohormone profile, and regulatory processes at the cellular level was also elucidated, with an emphasis on the interaction between stresses and hormonal cross-talk. We hope that these compiled articles will give a fresh, new insight into this topic, providing new ideas to pave the way for future research.

## Author contributions

All authors listed have made a substantial, direct and intellectual contribution to the work, and approved it for publication.

### Conflict of interest statement

The authors declare that the research was conducted in the absence of any commercial or financial relationships that could be construed as a potential conflict of interest.
